# B.P.F.C.® Bio-Plasma® with Pure Growth Factors (BioPlasma®) Used for the Treatment of a Persistent Great Periapical Lesion of an Endodontically Treated Tooth: A New Therapeutic Option

**DOI:** 10.1155/2020/4876437

**Published:** 2020-06-29

**Authors:** Raffaello Viganò, Mirko Disconzi, Edoardo Bertini, Luca Viganò, Cinzia Casu

**Affiliations:** ^1^DDS, Freelancer in Varese, Italy; ^2^University of Milano, Milano, Italy; ^3^Department of Oral Radiology, San Paolo Dental Building, University of Milano, Milano, Italy; ^4^Private Dental Practice, Cagliari, Italy

## Abstract

The aim of this case report was to evaluate the efficacy of a new platelet-rich plasma preparation and its regenerative capacity of bone periapical tissue for the treatment of a very compromised endodontic treated tooth, with a periapical lesion of 1.5 cm in diameter, using a pure platelet concentrate. This is made without the use of anticoagulant or any type of activator, e.g., bovine thrombin, calcium chloride. For this reason, it has been called “Pure”; it is the B.P.F.C.® Bio-Plasma® with Pure Growth Factors (BioPlasma®) designed and developed by Dr. Raffaello Viganò. The patient has read and signed a written consent form. The study protocol was approved by the Ethics Committee for Human Studies, University of Varese. X-ray at 2 and 6 months and 4 years after endodontic surgery demonstrated the success of the treatment.

## 1. Introduction

Recent literature reviews have shown that the survival of endodontically treated teeth is very high. Some studies reported that the success rate for endodontic surgery is between 82 and 94%, and it is considered an effective treatment before the replacement by a single-tooth implant [1, 2]. The majority of teeth with adequate root fillings, adequate restorations, and included in a recall program remained functional and healthy for more than 20 years [3]. However, around 10% of the patients who had an endodontic surgery 10 years before develop periapical lesions, due to several factors like missed canals, age, treatment sessions, and density of root filling [1]. Periapical lesions consistently undermine the tooth retention, suggesting subsequent extraction of the element.

Platelet concentrates (PCs) are biological autologous products obtained after various processing of a whole blood sample, mostly through centrifugation, and consist mainly of great concentration of platelets and growth factors (GFs) [4, 5]. The objective of the processing is to separate the blood components in order to discard elements considered as not usable (mostly the red blood cells, heavy and easily separated) and to gather and concentrate the elements that may be used for therapeutic applications such as fibrinogen/fibrin, platelets, growth factors, leukocytes, and other forms of circulating cells, in solution in liquid plasma [5]. In short, these products are extracts of the blood circulating tissue. They are tissues themselves and not pharmaceutical preparations. PCs are used on a surgical or wounded site in order to stimulate, improve, and accelerate healing [5]. In all wounds, the coagulation of blood to form a fibrin/platelet clot and matrix is the initial step of the natural healing process. The use of platelet concentrates was designed to reinforce this natural process. With time, this concept of optimization of healing evolved to a more sophisticated concept of tissue regeneration promoted by the growth factors and the cells contained in these preparations [5].

In modern surgery, PCs seem to enhance bone and soft tissue healing in alveolar ridge augmentation, periodontal surgery, socket preservation, implant surgery, endodontic regeneration, sinus augmentation, bisphosphonate-related osteonecrosis of the jaw (BRONJ), osteoradionecrosis, closure of oro-antral communication (OAC), and oral ulcers [4].

There are two main types of PCs, platelet-rich plasma (PRP) and platelet-rich fibrin (PRF), that contain anticoagulant citrate and the activation takes place with calcium chloride [5, 6]. There are several subclassifications of them.

Leukocyte-poor platelet-rich plasma products are preparations without leukocytes and with a low-density fibrin network after activation.

Leukocyte- and platelet-rich plasma (L-PRP) products are preparations with leukocytes and with a low-density fibrin network after activation.

Leukocyte-poor platelet-rich fibrin products are preparations without leukocytes and with a high-density fibrin network.

Leukocyte- and platelet-rich fibrin (L-PRF) products are preparations with leukocytes and with a high-density fibrin network [5].

The PRF families had several applications in oral and maxillofacial surgery and, in general, are usable in other disciplines with interesting results, particularly for the treatment of skin wound ulcers [5].

This article proposes a case of very compromised tooth with a big periapical lesion treated with a pure platelet-rich plasma concentrate, used to regenerate the bone. The aim of the study is to report the regenerative ability of this platelet concentrate, called Bio-Plasma®, described for the first time by Dr. Raffaello Viganò [6], and to show its possible use as a bone healing promoter in those cases where the bone and soft tissues have undergone a resorption process.

## 2. Case Presentation

**Figure 1:**
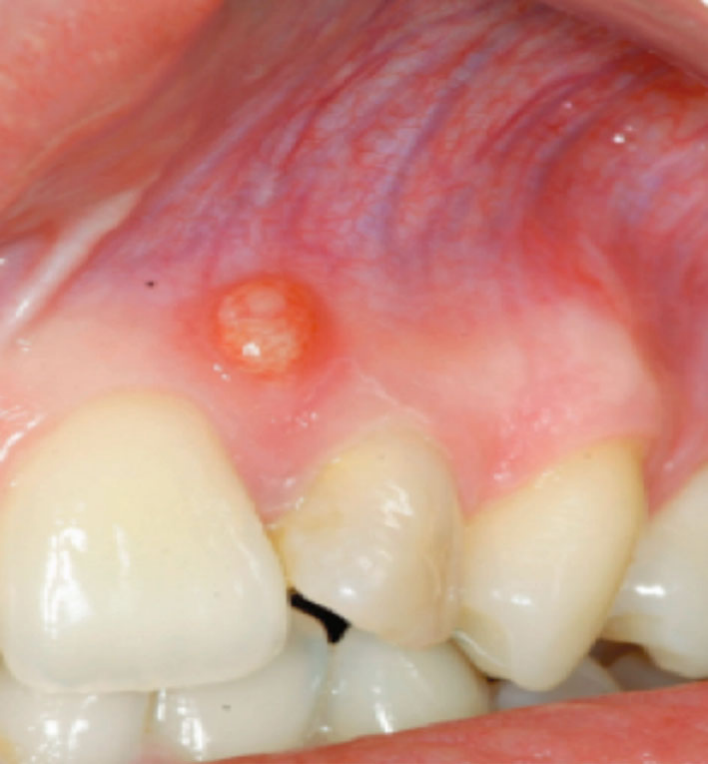
Starting clinical situation.

**Figure 2:**
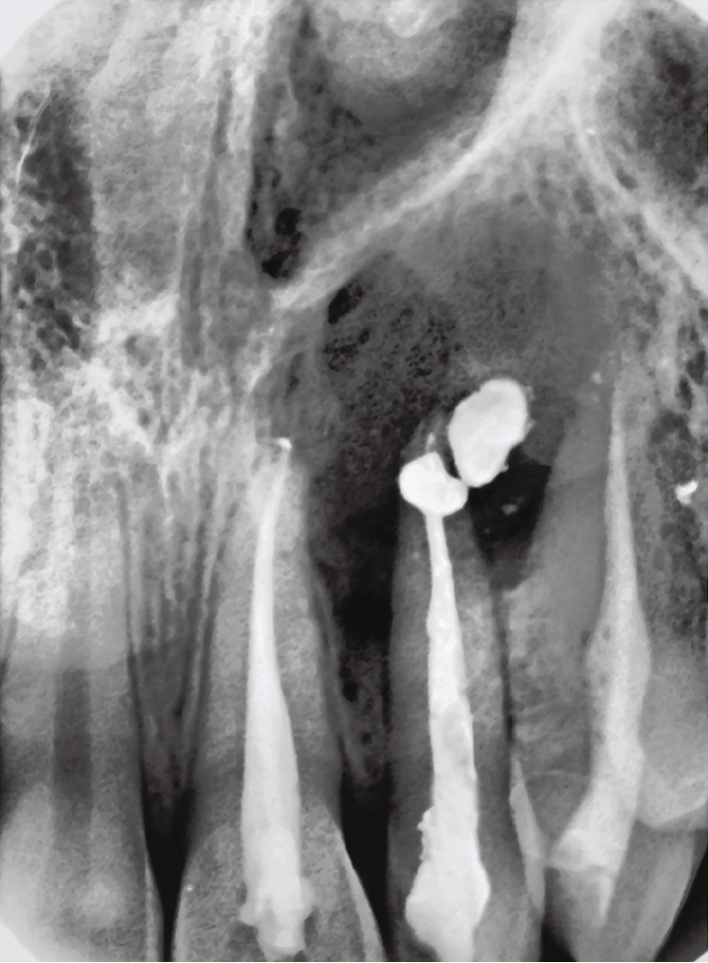
Starting X-ray.

**Figure 3:**
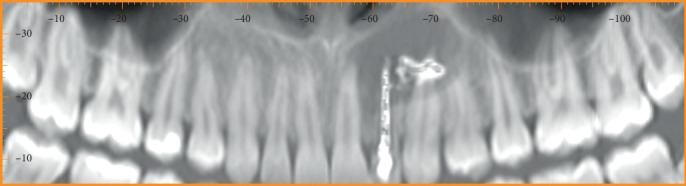
Starting CT.

A 20-year-old female patient had come to our attention for the evaluation of the previously treated element 2.2. She reported good health without systemic disease. The patient was submitted 12 months earlier to an endodontic treatment because element 2.2 was necrotic. A local big trauma could be the reason of the necrosis, but the patient was asymptomatic at the time when the necrotic tooth was discovered. She presented a fistula in correspondence of tooth 2.2 (Figure 1), and with an X-ray, it was possible to see the previous endodontic treatment and a radiolucent lesion greater than 1.5 cm in diameter (Figure 2). A computed tomography was performed (Figure 3). A diagnosis of periapical lesion was made. It was decided to maintain the dental element through an endodontic retreatment of the element by endodontic surgery with the positioning of the B.P.F.C. product. The patient has read and signed a written consent form. The study protocol was approved by the Ethics Committee of the Circolo Macchi Foundation Hospital of Varese with deliberative act no. 53 dated 08/02/2013.

**Figure 4:**
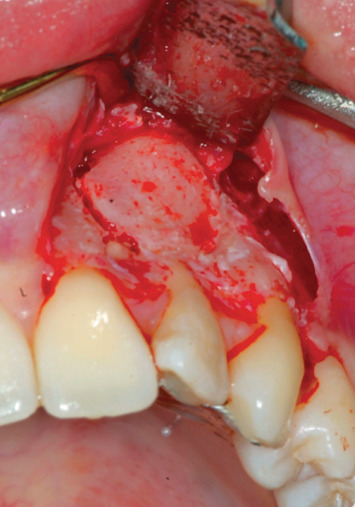
Preparation of the flap.

**Figure 5:**
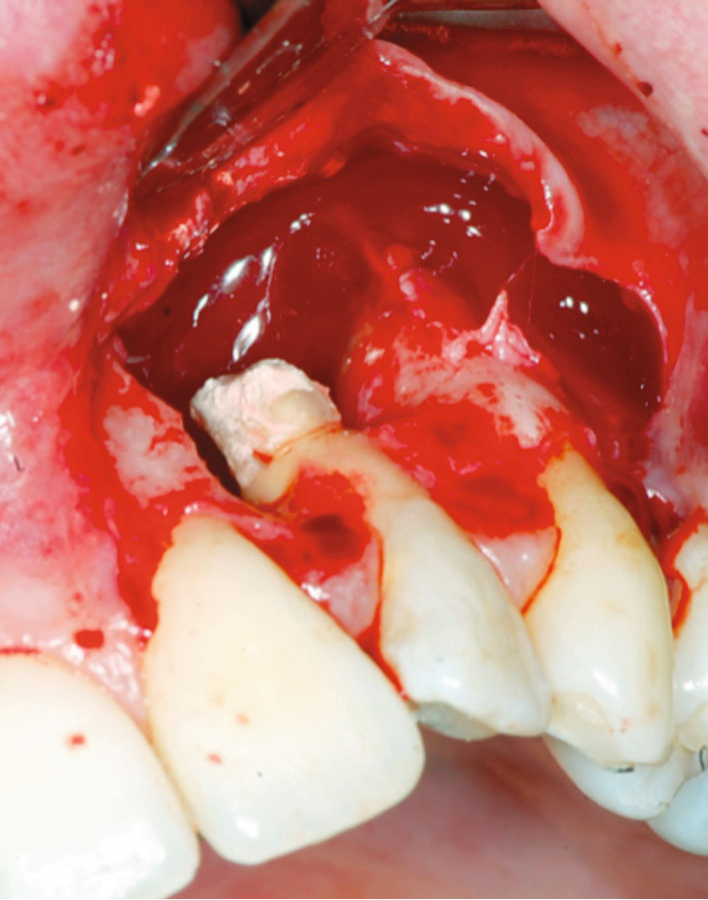
Endodontic surgery.

**Figure 6:**
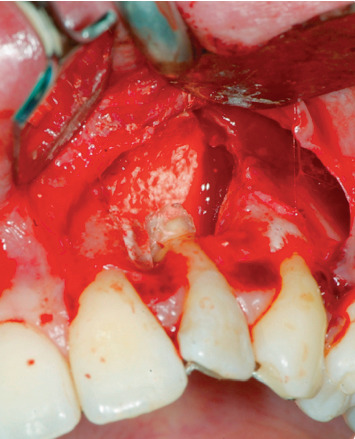
Bone curettage.

Endodontic retreatment was made with a retrograde endodontics. Local anesthesia was performed with mepivacaine with adrenaline 1 : 100,000, 1.8 ml with a 32 mm needle. A flap with 45 degree relief incision was performed. The incision was made with a number 15 blade. The flap produced had been carried out at partial thickness in order to ensure the greatest possible mobility (Figure 4). After removal of the inflammatory-infected material, a horizontal cut of the last 3 mm of the root apex had been performed with a diamond bur. K-files with sequence 15-20-25 taper 0.2 were used at first, then Pro file (Procad, Karlsruhe, Germany) from file 15 to file 40 (Figure 5). At the change of each instrument, irrigation with a solution of 5% sodium hypochlorite was performed. The root canal was closed with MTA (Dentsply Maillefer, Pennsylvania, USA) (Figure 6). After endodontic retreatment and curettage of the periapical site, the B.P.F.C. was prepared and inserted in the bone cavity.

**Figure 7:**
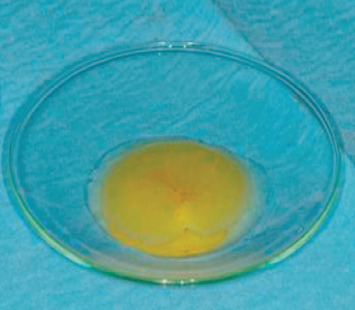
Membrane of a mixture of fibrin and poor plasma created.

**Figure 8:**
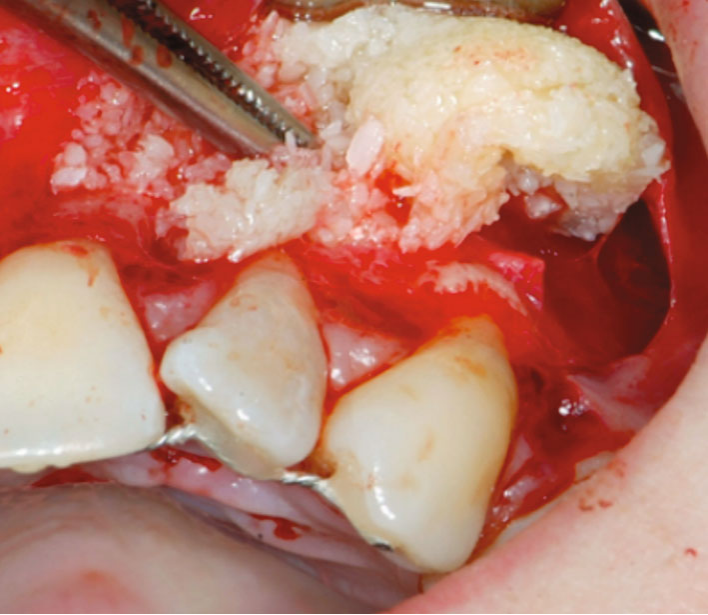
Positioning of the membrane.

**Figure 9:**
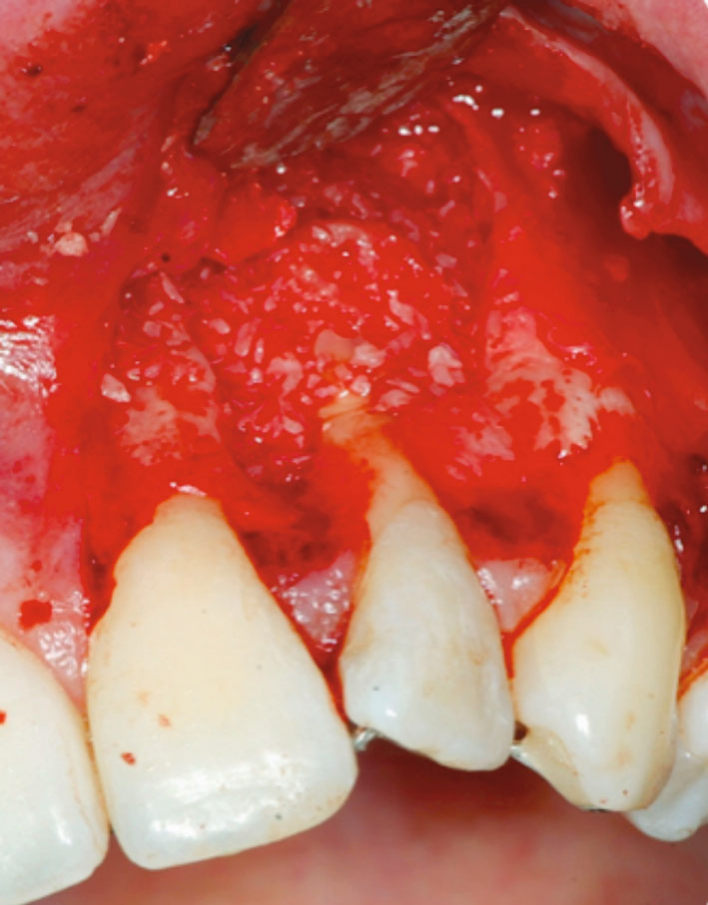
Membrane positioned.

**Figure 10:**
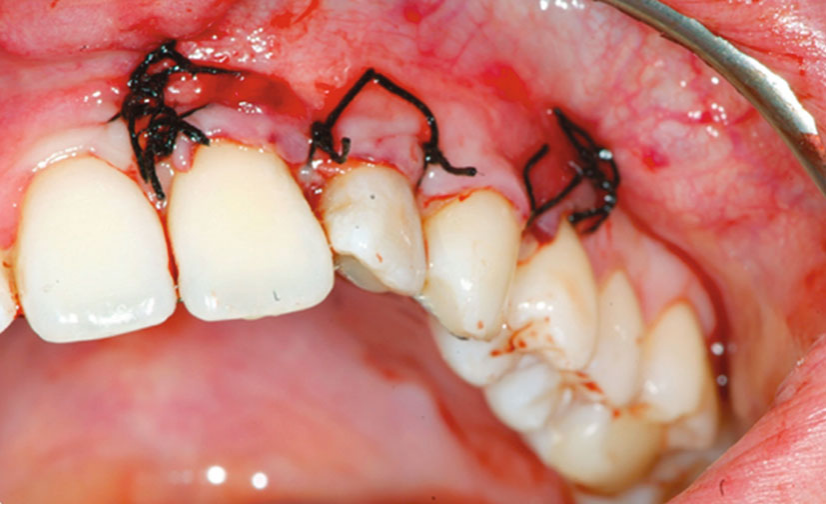
Final suture.

The phases of the protocol with B.P.F.C. were as follows:

1. 54 ml blood was taken from the patient by venepuncture of the antecubital vein
2. The blood was collected in 6 sterile Vacuette tubes (Greiner) with white cap; this indicates that they do not contain anticoagulant and/or subjected to any type of processing
3. Fractionation of the blood material: take 3 cc with 4/5 aspirations with a 500 *μ*l pipette and place the fraction obtained in the empty and numbered Vacuette. The protocol was provided for the elimination of leukocytes and red blood cells. This product was prepared with a special device designed and developed by Dr. Viganò
4. Creation of a membrane with a mixture of fibrin and poor plasma (the protocol does not include the use of any activator such as calcium chloride and bovine thrombin to obtain gelation) (Figure 7)
5. Preparation of rich gelled plasma and placement at the bone site (Figure 8)
6. Positioning of the membrane above the rich plasma (Figure 9)
7. Final suture (Vicryl wire 0000) (Figure 10)

**Figure 11:**
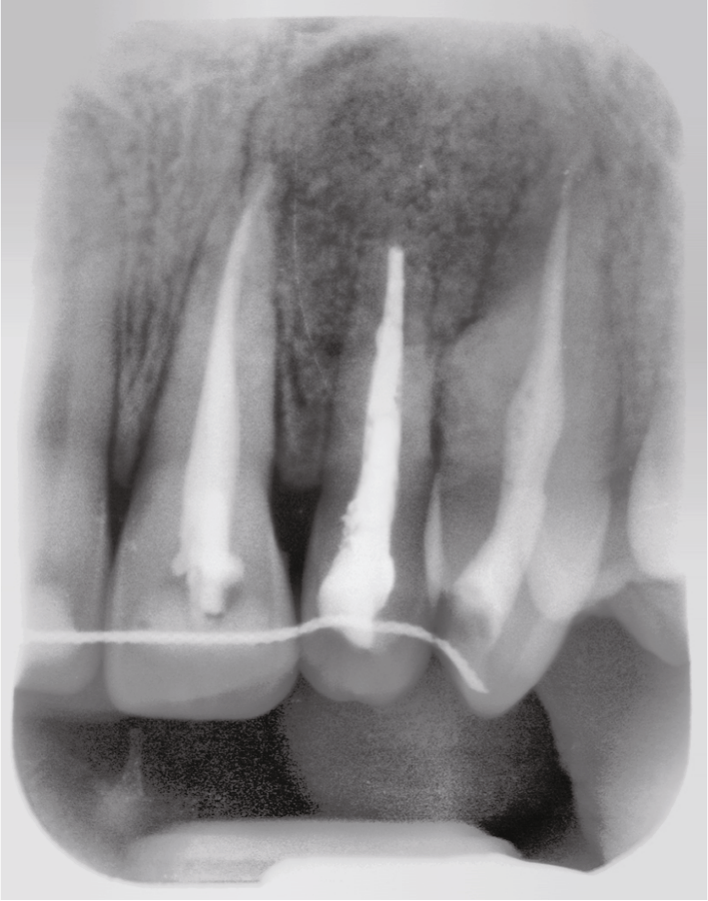
Postoperative X-ray.

It is important to underline that to prepare the membrane we need a minimum of 2 cc of fibrin. We had transferred the contents into a glass sterile container according to the quantity and size of the membrane to be obtained. This has to stand for 10-15 minutes on the thermostat at room temperature or 20-30 minutes without thermostat, before to place it. An X-ray was performed (Figure 11).

**Figure 12:**
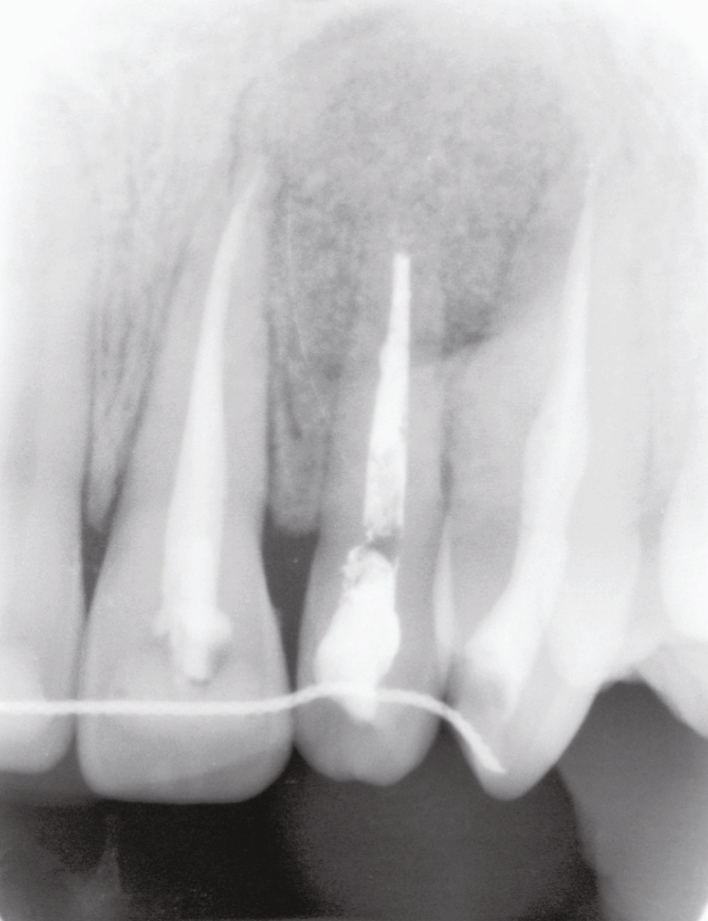
Healing after 3 months, clinical situation.

**Figure 13:**
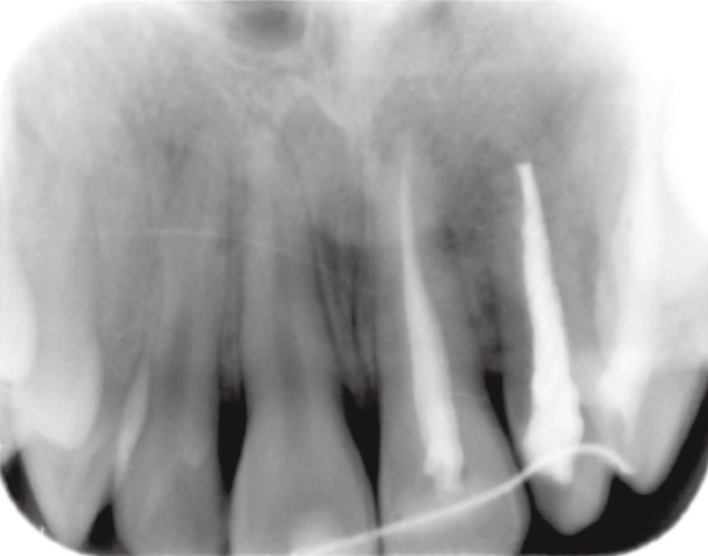
Healing at 6-month follow-up, X-ray.

**Figure 14:**
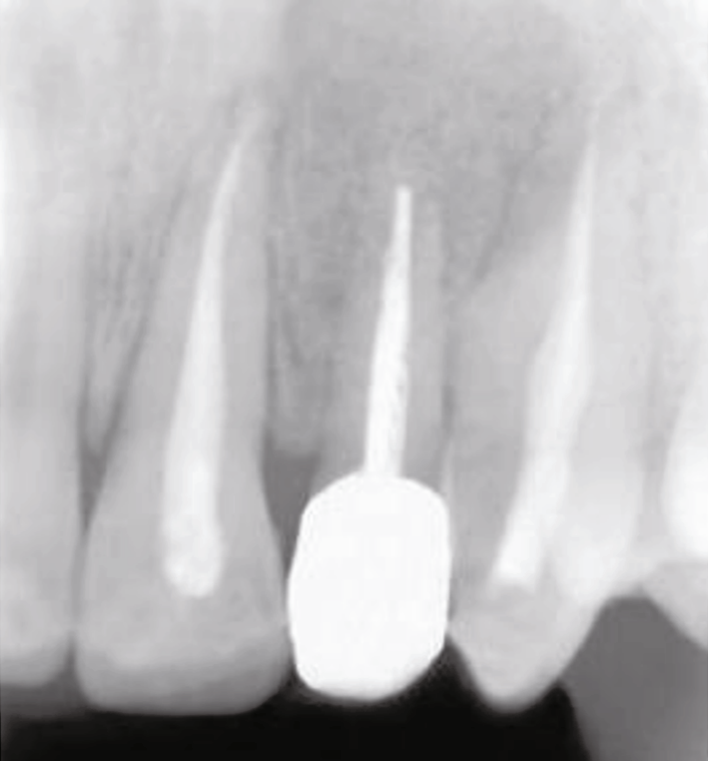
X-ray at 4-year follow-up.

After 2 months, the apical lesion completely healed (Figure 12). Follow-up at 2 and 6 months and 4 years demonstrated the success of the treatment (Figures 13 and 14).

## 3. Discussion

Our case report showed that the Bio-Plasma® with Pure Growth Factors, inserted in the bone cavity after removal of periapical lesion, could be an interesting therapeutic proposal for very compromised retreated elements instead of implantology.

The therapeutic objective of utilizing growth factors in oral surgery is to improve upon the body's regenerative capacity. In many situations, the unaided regeneration process is insufficient to allow complete repair of both bone and soft tissues [7].

With the advent of implantology, there is an increasing tendency to avoid dental retreatment of very complex elements and to prefer replacing it with a dental implant, but it is important to evaluate what are the prognoses of the two treatments (endodontics or implant surgery). The survival rate of dental implants varies from 91.8% to 100% after 1 to 10 years. No apparent differences on survival rates were noted depending on the time of placement. The quantitative synthesis of the success rate of zirconia implants was reported as 91.6% similar to that of titanium implants [8]. Sutter et al. in a very recent study (March 2020) reported that at the 1-year follow-up, 91.4% of the 81 teeth included in this retrospective study exhibited successful clinical and radiographic healing. They concluded that the type of tooth was significantly associated with the success of the surgery, but not other variables such as radiological severity of periapical inflammation, lesion histopathology, administration of antibiotics, smoker status, the quality of the root canal treatment, and preoperative pain and clinical signs of inflammation [9]. Alghamdi et al. in their review found that in previous works authors stressed that the healing of periapical lesions after endodontic surgery is faster than other surgery treatment, although it is very difficult to make a comparison with different works in the literature which report different follow-up times [10]. For this reason, the endodontic surgery seems better for our case. To improve the speed of healing of endodontic lesions, reduce postoperative symptoms, and improve bone quality, several researchers have tried to combine devices such as laser and photodynamic therapy with endodontic surgery, obtaining good results. However, there are still too few studies on these fascinating principals [11, 12].

The use of PRF to improve the healing of endodontic lesion was successfully documented in recent literature by Popowicz et al., with 2 case reports at 1-year follow up [13], and also by Goyal et al. with 3 case reports in a study in March 2020 [14]. However, in larger studies where the use of PRP was added to endodontic surgery, the difference in terms of bone quality compared to that of the control group was not statistically significant [15]. Betancourt et al. used other platelet derivatives for this purpose such as L-PRF on a case of severe endoperiodontal injury, in which there was an important improvement only from the periodontal point of view [16]. The long-term success of endodontic surgery is high, and it is currently preferable to tooth extraction and implant positioning. However, healing for damage greater than 1 cm takes several months [1, 2], while in our case it was already visible at 2 months. A good curettage of the residual bone cavity, the instrumentation, and the seal with MTA have certainly helped to determine an improvement in bone quality and speed of healing. The properties of the MTA are widely supported by several articles in the most recent review [17]. The limits of this protocol are related to the formation of a prepared team (assistant, nurse, etc.) and the patient's fear of needle. Several studies demonstrate how a well-trained staff is a strong predictable factor for the outcome of medical treatments [18].

Further clinical trials are needed to validate this starting result.
